# An assessment of bioinformatics tools for the detection of human endogenous retroviral insertions in short-read genome sequencing data

**DOI:** 10.3389/fbinf.2022.1062328

**Published:** 2023-02-08

**Authors:** Harry Bowles, Renata Kabiljo, Ahmad Al Khleifat, Ashley Jones, John P. Quinn, Richard J. B. Dobson, Chad M. Swanson, Ammar Al-Chalabi, Alfredo Iacoangeli

**Affiliations:** ^1^ Department of Basic and Clinical Neuroscience, King’s College London, Maurice Wohl Clinical Neuroscience Institute, Institute of Psychiatry, Psychology and Neuroscience, London, United Kingdom; ^2^ Department of Biostatistics and Health Informatics, King’s College London, Institute of Psychiatry, Psychology and Neuroscience, London, United Kingdom; ^3^ Department of Pharmacology and Therapeutics, Institute of Systems, Molecular and Integrative Biology, University of Liverpool, Liverpool, United Kingdom; ^4^ NIHR Biomedical Research Centre at South London and Maudsley NHS Foundation Trust and King’s College London, London, United Kingdom; ^5^ Institute of Health Informatics, University College London, London, United Kingdom; ^6^ NIHR Biomedical Research Centre, University College London Hospitals NHS Foundation Trust, London, United Kingdom; ^7^ Department of Infectious Diseases, School of Immunology and Microbial Sciences, King’s College London, London, United Kingdom; ^8^ Department of Neurology, King’s College Hospital, London, United Kingdom

**Keywords:** herv-k, benchmarking, whole-genome sequencing, retrovirus, bioinformatics

## Abstract

There is a growing interest in the study of human endogenous retroviruses (HERVs) given the substantial body of evidence that implicates them in many human diseases. Although their genomic characterization presents numerous technical challenges, next-generation sequencing (NGS) has shown potential to detect HERV insertions and their polymorphisms in humans. Currently, a number of computational tools to detect them in short-read NGS data exist. In order to design optimal analysis pipelines, an independent evaluation of the available tools is required. We evaluated the performance of a set of such tools using a variety of experimental designs and datasets. These included 50 human short-read whole-genome sequencing samples, matching long and short-read sequencing data, and simulated short-read NGS data. Our results highlight a great performance variability of the tools across the datasets and suggest that different tools might be suitable for different study designs. However, specialized tools designed to detect exclusively human endogenous retroviruses consistently outperformed generalist tools that detect a wider range of transposable elements. We suggest that, if sufficient computing resources are available, using multiple HERV detection tools to obtain a consensus set of insertion loci may be ideal. Furthermore, given that the false positive discovery rate of the tools varied between 8% and 55% across tools and datasets, we recommend the wet lab validation of predicted insertions if DNA samples are available.

## 1 Introduction

**FIGURE 1:**
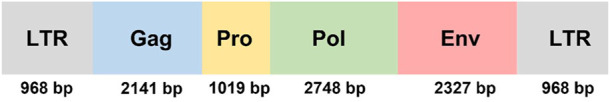
This schematic representation shows the general structure of a full length HERV-K with approximate base pair lengths for each section. The LTR length is the LTR5_Hs length given in the DFAM database (accession = DF0000558), the internal gene lengths come from HERVK11 internal regions (DFAM accession = DF0000189) (Storer et al., 2021). The gag gene encodes a polyprotein that is cleaved to produce the structural proteins that make up a viral particle. The pro gene encodes the viral protease while pol codes for the viral enzymes required for reverse transcription and integrase. The env gene codes for the viral envelope protein that mediates viral entry into a target cell. The gene products require post translational cleavage, for example, pro cleaves the gag polyprotein into “matrix”, “capsid” and “nucleocapsid” subunits. The majority of HERV-K loci only contain the LTR regions which contain the enhancer and promoter sequences that regulate transcription.

Endogenous retroviruses (ERVs) integrated into the genome of vertebrates as a result of ancient exogenous infections. They invaded the germ cell lines of all vertebrates including humans, becoming an integral part of the germline transmission and therefore replicate in a Mendelian fashion (Gifford and Tristem, 2003). Human endogenous retroviruses (HERVs) comprise ∼8% of the genome, whereas protein coding genes comprise only 1%–2% (Pisano et al., 2019). Although they make up a striking portion of the human genome, most of them are inactive as a consequence of the accumulation of mutations and DNA methylation (Belshaw et al., 2005). The HML-2 HERV-K subgroup includes some of the most recent HERV integrations, which are found as full length (or near full length) sequences in over 80 different loci (Subramanian et al., 2011). Though there are several full-length copies of HERV-K in the genome, none are likely to produce an infectious virus (Boller et al., 2008). HERV-K sequences can be full length proviruses, solo long terminal repeats or 2-LTR sequences and are polymorphic in the human population (Garcia-Montojo et al., 2018). A full length ERV provirus consists of long terminal repeats (LTRs) flanking the viral genes (gag, pro, pol and env). In the majority of elements defined as HERV loci, only the LTRs are present and these contain the promoter and enhancer regions (Klaver and Berkhout, 1994) (Figure 1).

HERVs are classified as transposable elements (TEs) but there are notable differences between HERVs and other TEs. HERVs are less common than Alus and LINEs (long interspersed nuclear elements) and a full length HERV-K provirus is approximately 10 kb in length, compared to full length LINEs which are 6 kb in length, while Alu insertions are approximately 300 bp (Payer and Burns, 2019). LINEs are highly capable of transposition and Alus can hijack LINE mechanisms to the same end, in fact Alus are the most abundant transposable element in the human genome. In contrast to this, HERVs generally lack transposon function, though they can transcribe RNA (Dolei et al., 2019). A further difference is that HERVs are flanked by LTR sequences, containing promoter and enhancer regions, which are not found in LINEs or Alus (Larsen et al., 2018). The SVA (Sine VNTR Alu) is another TE reliant on LINE1 for transposon function and is a composite TE containing ALU and HERV LTR sequence. In contrast to HERVs, most SVA elements are full length while the majority of HERVs contain only the flanking LTRs (Hancks and Kazazian, 2010).

Characterizing the HERV genomic landscape is challenging. HERVs are thousands of bases long and highly repetitive. This means that short-read genome sequencing cannot characterize the sequence of HERVs that are not present in the reference genome, beyond a limited number of bases (Ewing, 2015). Furthermore, reads from repetitive regions can introduce ambiguities in the mapping step of genome alignment where there are multiple putative matches (Teissandier et al., 2019). This issue extends to biological tests of transposable elements, as short oligos designed to target a specific TE locus may sit down at multiple locations on the genome (Bourque et al., 2018).

HERV-Ks, and related transposable elements, have been linked to a wide range of diseases including cancer and neurodegenerative diseases, such as amyotrophic lateral sclerosis (ALS), *via* multiple mechanisms. For example, their insertion into the human genome may alter gene expression or disrupt reading frames (Buzdin et al., 2003); they were reported to be upregulated in biological samples from people affected by neurodegenerative diseases and cancer (Garcia-Montojo et al., 2020; Dervan et al., 2021; Jones et al., 2021); furthermore, their expression may be toxic for certain cell types such as motor neurons (Li et al., 2015). Given their proposed broad role in human diseases, we focused our work on HERV-Ks.

Recent advances in next-generation sequencing (NGS) have made sequencing large DNA molecules a common practice in genetic research, allowing for the investigation of a wide range of variants from single nucleotide variants to large structural variants (Iacoangeli et al., 2019a). This technology has also been used to study HERVs (Xue et al., 2020a). It is established that HERVs can express RNA which can be captured in NGS experiments. For example, RNA sequencing experiments have quantified HERV RNA in healthy and tumor cell lines (Rezaei et al., 2021) and have highlighted HERV RNA as a biomarker for cell pluripotency (Santoni et al., 2012). Chip-seq experiments have highlighted a role for HERV-H loci in chromatin restructuring during cell differentiation (Zhang et al., 2019). Genome wide methylation studies may prove to be very useful in understanding HERV regulation as hypomethylation of HERVs correlates with increased expression (Chiappinelli et al., 2015).

**FIGURE 2:**
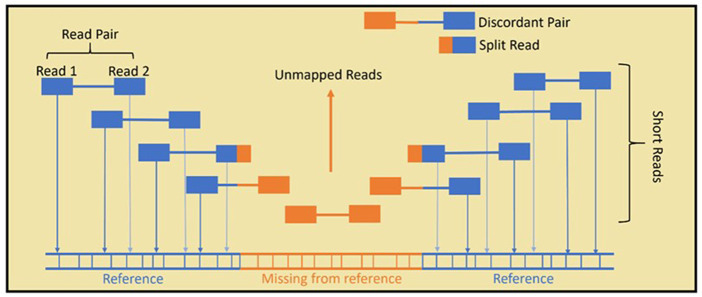
In standard Illumina paired-end SR-WGS, the DNA sample is first sheared into small fragments. Then, both ends of the fragment are sequenced, giving paired reads. Read mapping is achieved by aligning the reads to a reference genome. HERVs that are present in the sample but not the reference, cannot be aligned fully and remain largely unmapped, which means they are often overlooked in genome analysis. Specialised bioinformatics tools use unmapped, split and discordant reads to predict non-reference HERVs.

Currently, a number of bioinformatic tools for the identification of HERV insertion loci in short-read whole-genome sequencing (SR-WGS) data exist, mostly based on the exploitation of split and discordant reads to reveal the presence of potential HERV insertions (Figure 2). Some of these tools have been developed to detect a range of TEs, including HERVs, Alus and LINEs, while others are specifically targeted to HERV detection. Given the lack of a comprehensive and independent assessment of their performance, an evaluation of the current tools for HERV detection is greatly needed for the design of optimal analysis pipelines and to promote the discussion necessary for the scientific community to establish best practice protocols. On this basis, we designed a set of experiments to benchmark widely used computational tools and protocols for the detection of HERVs in SR-WGS data (short reads = 50–200 bp). We hypothesize that specialist HERV tools might perform better than general TE detectors and we aim to quantify the benefits and limitations of using generalist or specialized tools to study HERVs in NGS. Considering their proposed role in human disease, we focused our experiment on the identification of HERV-K insertions that are not present in the human reference genome (non-reference HERV-Ks). We tested six widely used tools on three short-read sequencing datasets: a large short-read whole-genome sequencing (SR-WGS) dataset (50 human samples), a simulated SR-WGS dataset, and six SR-WGS samples for which matching long-read sequencing data was available.

## 2 Methods

### 2.1 Overview of the tested tools

MELT: MELT scans WGS data for clusters of discordant read pairs and split reads. Split and discordant reads can be mapped to an insertional element reference sequence provided by the user, to allow the detection of specific insertion types. It can also genotype reference mobile elements (Gardner et al., 2017). MELT has previously been used to integrate TE insertion and TE expression data in cancer lines (Clayton et al., 2016) and to elucidate the evolutionary mechanisms underlying TE diversity (Rishishwar et al., 2018). According to its documentation, MELT was not tested for HERV detection in its original publication, and the authors predicted that it may perform poorly on LTR elements compared to non-LTR transposable elements (such as Alus). Nevertheless, MELT has been used to detect HERVs (Santander et al., 2017; Chen and Li., 2019; Feusier et al., 2019).

Mobster: This tool also uses discordant reads alongside split reads and an insertional reference sequence to predict specific insertion sites. Mobster has also been used to highlight the role of TEs in cancer (Clayton et al., 2016) and has been used to show an association between TEs and autism (Borges-Monroy et al., 2021). When Mobster was released, the authors reported that it was not able to identify HERV insertions. However, they tested it using just two WGS paired-end samples and since its first publication in 2014, Mobster has been extensively upgraded and gained a considerable popularity. We included it in our experiment, tested its updated version on a larger sample and used a different HERV-K template sequence to that used in the authors’ benchmarking work (Thung et al., 2014).

Retroseq: Retroseq uses discordant read pairs to identify putative insertion sites and filters for read pairs which align to a reference of interest (Keane et al., 2013). Retroseq has been used for mapping transposable elements in evolutionary studies (Dennenmoser et al., 2019) and extensively adopted as the starting point of more advanced pipelines (Chen and Li, 2019).

Steak: Steak annotates both reference and non-reference mobile elements. Unlike the other tools, it first identifies reads that partially map to the target HERV reference sequence. These reads are assumed to map to the edge of the insertion. The mapped fragments of the reads are removed, and a library of host reference flank reads and their mates is created. These reads are mapped onto the human reference genome to identify the presence of both reference and non-reference HERV loci (Santander et al., 2017). Steak has been less widely used than MELT, Mobster and Retroseq, though it has been used in combination with PCR amplification to map HERV-K loci in the genome (Xue et al., 2020b) and is regularly used as a benchmarking comparison for new tool publications.

ERVcaller: The tool extracts incorrectly mapped read pairs and split reads to identify likely insertions sites, that are then aligned to a reference sequence to allow for the detection of specific insertion types (Chen and Li, 2019). ERVcaller has been used in combination with chip-seq and RNAseq analysis to quantify the contribution of TEs to epigenetic regulation (Groza et al., 2022).

Retroseq+: This pipeline is our in-house implementation of a protocol described by Wildschutte et al. It uses Retroseq as a base for predicting HERV-K insertion loci. It then refines the results through insertion junction reconstruction and secondary scanning of the junction for HERV-K sequence by RepeatMasker. Because this protocol is not available as an automatic bioinformatics pipeline, we have implemented it ourselves, following the authors’ description (Wildschutte et al., 2016; Kabiljo et al., 2022).

These chosen tools include widely used, established TE detectors (Mobster, MELT and Retroseq) as well as newer tools specifically developed for HERV detection (ERVcaller, Steak, Retroseq+). We did not include tools designed for the analysis of tumor cell lines, or tools designed to analyze data other than short-read NGS, or evolutionary aspects of HERVs as they fell outside our scope to test tools for the detection of germline non-reference HERV-K insertions in short-read NGS data. Scripts for these tools are available as supplementary materials as are flow diagrams explaining each tool in more detail.

### 2.2 Benchmarking experiments overview

In order to assess the performance of these tools, we set up four experiments: i) we estimated the performance of the tools using simulated NGS data; ii) using the HERV-K calls from the 50 SR-WGS samples, we attempted to validate the tools by quantifying the proportion of predicted HERV-K insertions that were previously reported in literature; iii) using the tools on 50 SR-WGS samples, we measured the agreement between tools; iv) finally, we assessed the specificity of each tool by using long-read data for the validation of HERV-K calls from matching short-read data.

### 2.3 Simulated short-read WGS analysis

The purpose of this test was to assess the sensitivity and specificity of each tool using simulated data with a known set of insertions. For the simulation test, short-read paired-end Illumina WGS data were simulated from the hg19 reference sequence using DWGSIM (parameters in Table 1) (Homer, 2010). Hg19 is reported to have 66 HERV-K (HML-6) full length, proviral loci (2) from which we randomly selected 15 of type LTR3A to use as target LTRs (Supplementary File S4). Furthermore, in order to test whether the tools are able to distinguish between LTR types, we also randomly selected four LTR3B type HML-6 proviral loci (Supplementary File S5). Both LTR3A and LTR3B are HML6 insertions but have been shown to cluster separately in phylogenetic analyses (Pisano et al., 2019). We expect that including both subtypes in our test may provide a higher degree of resolution into the evaluation of the tools’ accuracy.

**TABLE 1:** Custom parameters used to generate the simulated data. All parameters not included in this table were kept as default.

	Read length	No. reads	Coverage	Outer distance	-e (error)	-s (std-dev outer dist)
Genome 1	150 bp	333,333,333	32X	400	0.020	5
Genome 2	100 bp	500,000,000	32X	400	0.020	5
Genome 3	150 bp	105,000,000	10.5X	400	0.020	5
Genome 4	100 bp	105,000,000	7X	400	0.020	5

To simulate these HERV-K sites as novel insertions, after generating the WGS data, we removed these proviruses from the hg19 reference using Bedtools masking followed by deletion of the mask (Quinlan and Hall, 2010). The simulated FASTQ files were then aligned to the edited hg19 using BWA-MEM. Thus, the simulated data contained 19 known HERV-K insertions that were not present in our edited reference genome, and these insertions were the only non-reference HERV elements in the simulated samples. Each tool was then applied to the simulated WGS. Only the LTR3A sequence was used as target reference sequence template, meaning each tool should have only detected the 15 LTR3A loci, not the 4 LTR3B loci. This allowed us to see how well each tool can distinguish specific insertion types as well as assess general sensitivity. We defined sensitivity as the proportion of the known, non-reference insertions which were successfully detected: True positives/(True positives + False negatives). We defined precision as the proportion of positive results that are true: True positives/(True positives + False positives).

### 2.4 Overlap analysis and comparison with previously reported HERVs

Each tool was applied to WGS data of 50 ALS patients from the British Project Mine dataset (Project MinE ALS Sequencing Consortium, 2018; Iacoangeli et al., 2019b). This WGS was generated from blood samples, using the Illumina Hiseq 2000 platform. The resulting WGS samples had read length equal to 100 bp with an average coverage depth of 40X (paired-end reads). We aligned them to the hg19 reference genome using Burrows-Wheeler alignment, BWA-MEM (Li, 2013). The predicted insertion sites across all genomes were compared to a list of 40 well characterized polymorphic HERV-K insertions previously described in the literature (Supplementary File S1) (Kahyo et al., 2017). They were also compared to all reference HERV loci (both HERV-Ks and other HERV/LTR subgroups) obtained through the UCSC table browser using the RepeatMasker (RMSK) track for the hg19 genome build. The following identifiers were used to retrieve HERV-K reference loci: LTR5_Hs, LTR5A, LTR5B, HERV-K and HERV-K-int (Supplementary File S2); while the set of all reference HERVs was obtained by taking the entire UCSC RMSK hg19 track and extracting those which had the identifiers “ERV” or “LTR” (Supplementary File S3). HML-2 type HERV-Ks, which are targeted in this analysis can be subclassified based on their LTR sequence. LTR5B is the phylogenetically oldest HML-2 LTR, LTR5_Hs is younger and human specific.

**FIGURE 3:**
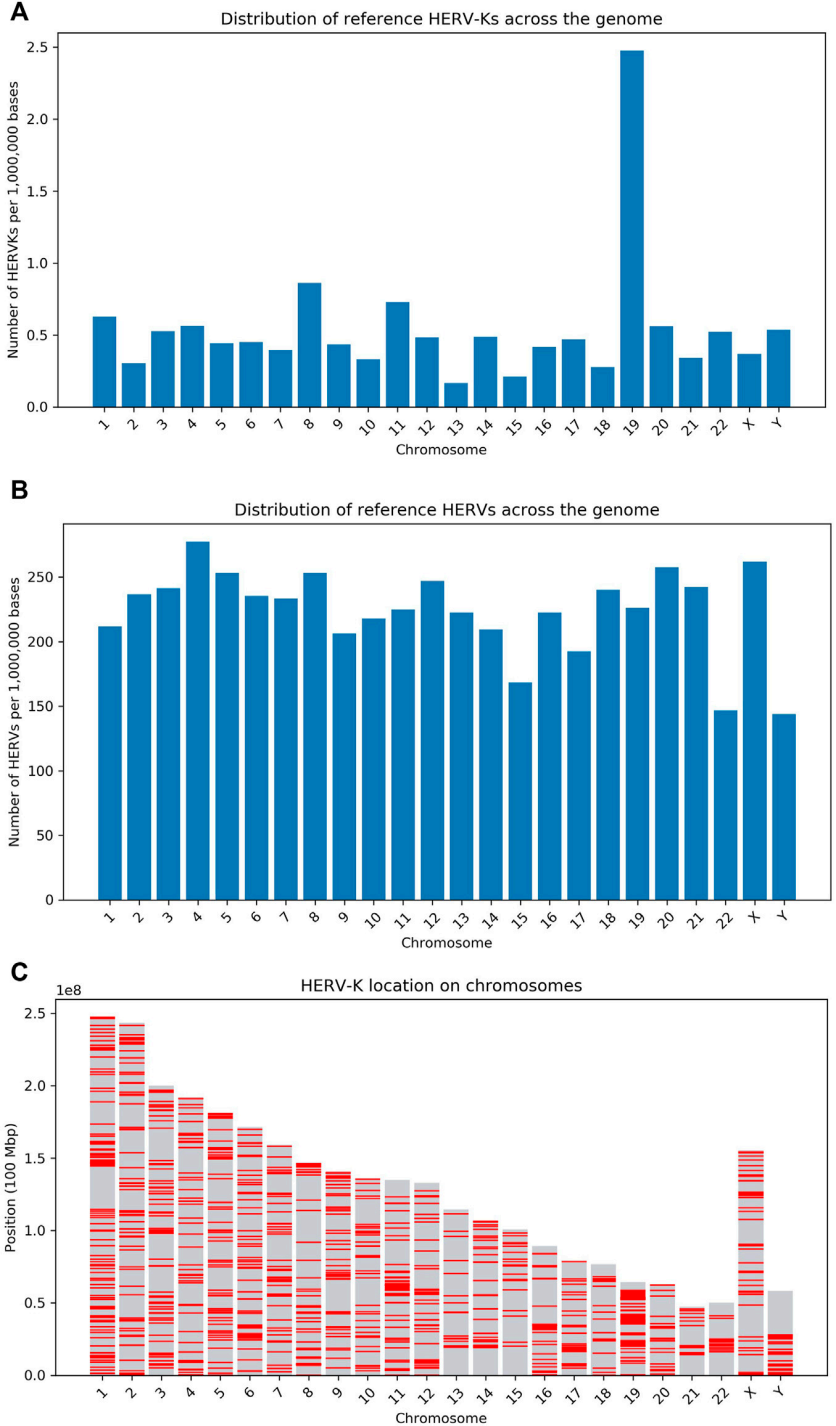
Overview of the density of HERV loci in the human genome. **(A)** Number of HERV-Ks per million bases in each human chromosome (as given by the UCSC RMSK table). The high HERV density in chr19 has been previously reported (Katzourakis et al., 2007) and other transposable elements are also enriched on this chromosome (Gianfrancesco et al., 2019). **(B)** Number of HERVs and LTR sequences per million bases in each human chromosome–Data is from the UCSC RMSK table. **(C)** This panel shows the distribution of HERV-K on each chromosome, with a red line indicating the presence of the HERV-K LTR. These results are obtained from the LTR5/HERV-K UCSC RMSK table.

An overlap was defined as a predicted insertion being within 500 base pairs of a known ERV locus. The number of reference HERV-Ks and HERVs per million bases across the human chromosomes is shown in Figures 3A–C.

This test allowed us to quantify the proportion of predicted insertions of each tool that matched known and validated HERV-Ks under the assumption that such calls are more likely to be true positives and therefore tools which show a higher proportion are more reliable.

The results of each tool were also compared to one another, giving for each one of them, the proportion of its results that were also predicted by each of the other tools. For Steak and Melt reference results were filtered out from the total results using the UCSC RMSK table of hg19 reference HERV-K loci (Supplementary Table S2). This allowed us to quantify the agreement across tools.

### 2.5 Long-read sequencing data

We used a set of six samples from Wang et al., for which both short and long-read genome sequencing data were available (Wang et al., 2019) (GIAB data, IDs: HG002, HG003, HG004, HG005, HG006, HG007). Briefly, the short-read data (derived from blood) were sequenced using Illumina Hiseq 2500 giving mean 105 bp paired end reads with coverage depth ranging between 15.6X and 18.8X. The long reads were sequenced using PacificBio Sequel system version 2. For these samples the read lengths were between 10 KB and 18 KB and the mean coverage depth of samples ranged between 28.5X and 69X.

Long-read sequencing (read length >10,000 base-pairs) can capture a large overhang, if not the whole, of the HERV-K allowing for their accurate identification (Chu et al., 2021; Chu et al., 2021; Troskie et al., 2021). We applied each tool to the short-read WGS data to predict LTR5_Hs HERV-K insertions and used the long-read data for validation as follows. For each predicted insertion, we extracted long reads mapped at the corresponding locus from the matching long-read WGS sample. These long reads were then assembled into contigs using wtdbg2 (Ruan and Li, 2020). RepeatMasker (Tempel, 2012) can detect and classify repetitive elements in genomic sequences. It was applied to the long-read assembled contigs to confirm the presence of the HERV-K LTR5_Hs sequence at each predicted locus.

If the contig, at a given locus where a short-read based prediction was made, tested positive for HERV-K when analysed with RepeatMasker, the predicted HERV-K insertion was considered true. The proportion of each tool’s predictions which are successfully validated is an indicator of the tool accuracy.

### 2.6 Computational efficiency report

The computational efficiency of each tool is an important factor, especially if the users have limited resources and large datasets. The purpose of this test was to quantify the computational resources required by each tool.

We tested the memory usage and time taken for each tool to run on a single WGS sample from the Project MinE dataset. Slurm was the scheduling system on the Linux HPC platform used for this project. Slurm has its own command (sacct) for timing scripts and assessing memory usage. Each tool was applied to a single WGS sample from Project MinE and sacct was used to report the tools memory and cpu usage. To determine the sizes of intermediate files produced by each tool, the “du” command was run on a loop, executing every second, over the directory in which each tool was running. The difference between the starting directory size and largest directory size reported by “du” is reported.

## 3 Results

### 3.1 Simulated data results

Each tool was applied to a set of four simulated WGS samples, with known HERV insertions of different types (LTR3A and LTR3B). Each tool was given LTR3A as target element. This allowed us to estimate the sensitivity and precision of each tool. Precision and sensitivity highly varied across tools, ranging between 0.56–0.92, and 0.20–0.80 respectively on the higher quality samples (32X and 150 bp reads). Although the best performing tool was ERVcaller, HERV specialist tools did not perform consistently better than generalist tools, and Steak showed the lowest precision and sensitivity across all simulated genomes. All tools performed worse on samples with lower read depth (Table 2). For example, Retroseq detected 11/15 LTR3A insertions in a 32X sample but only 7/15 LTR3A insertions in the 10.5X and 7X samples. However, the degree to which read depth affects each tool varied. ERVcaller performed equally well on 32X and 10.5X samples finding 12 of the 15 LTR3A insertions and, in the 7X sample, it still identified 10 insertions. The only tool to mistakenly detect an LTR3B insertion was Retroseq. Sensitivity and precision greatly varied across tools. E.g. Steak and ERVcaller had the highest average precision (0.9) while Retroseq+ had the lowest average precision (0.63). However, Steak had the lowest average sensitivity (0.13) while ERVcaller had the highest (0.77). Mobster did not detect any insertions in this experiment.

**TABLE 2:** Results of analysis of simulated WGS data. Each row corresponds to a different tool. The table reports the number of correctly identified LTR3A insertions (the target insertions, 15 loci in the simulated genome), the number of LTR3B insertions (4 loci in the simulated genome) mistakenly classified as LTR3A insertions, and the total number of predicted insertions, including the ones that did not correspond to any of the 19 simulated insertion loci, for each sample.

	Genome 1 (150 bp, 32X)					Genome 2 (100 bp, 32X)					Genome 3 (150 bp, 10.5X)					Genome 4 (100 bp, 7X)				
	LTR3A	LTR3B	Total	Precision	Sensitivity	LTR3A	LTR3B	Total	Precision	Sensitivity	LTR3A	LTR3B	Total	Precision	Sensitivity	LTR3A	LTR3B	Total	Precision	Sensitivity
Retroseq	11	2	16	0.68	0.73	11	2	18	0.61	0.73	7	0	8	0.88	0.46	7	0	8	0.88	0.46
Retroseq+	10	0	18	0.56	0.67	9	0	20	0.45	0.60	8	0	12	0.67	0.53	5	0	6	0.83	0.33
Melt	7	0	9	0.78	0.46	7	0	9	0.78	0.46	8	0	9	0.89	0.53	7	0	9	0.78	0.46
Steak	3	0	5	0.6	0.20	2	0	2	1.00	0.13	2	0	2	1.00	0.13	1	0	1	1.00	0.07
ERVcaller	12	0	13	0.92	0.80	12	0	14	0.86	0.80	12	0	13	0.92	0.80	10	0	11	0.91	0.67
Mobster	0	0	0	0	0	0	0	0	0	0	0	0	0	0	0	0	0	0	0	0

### 3.2 Analysis of the 50 short-read WGS samples

**FIGURE 4:**
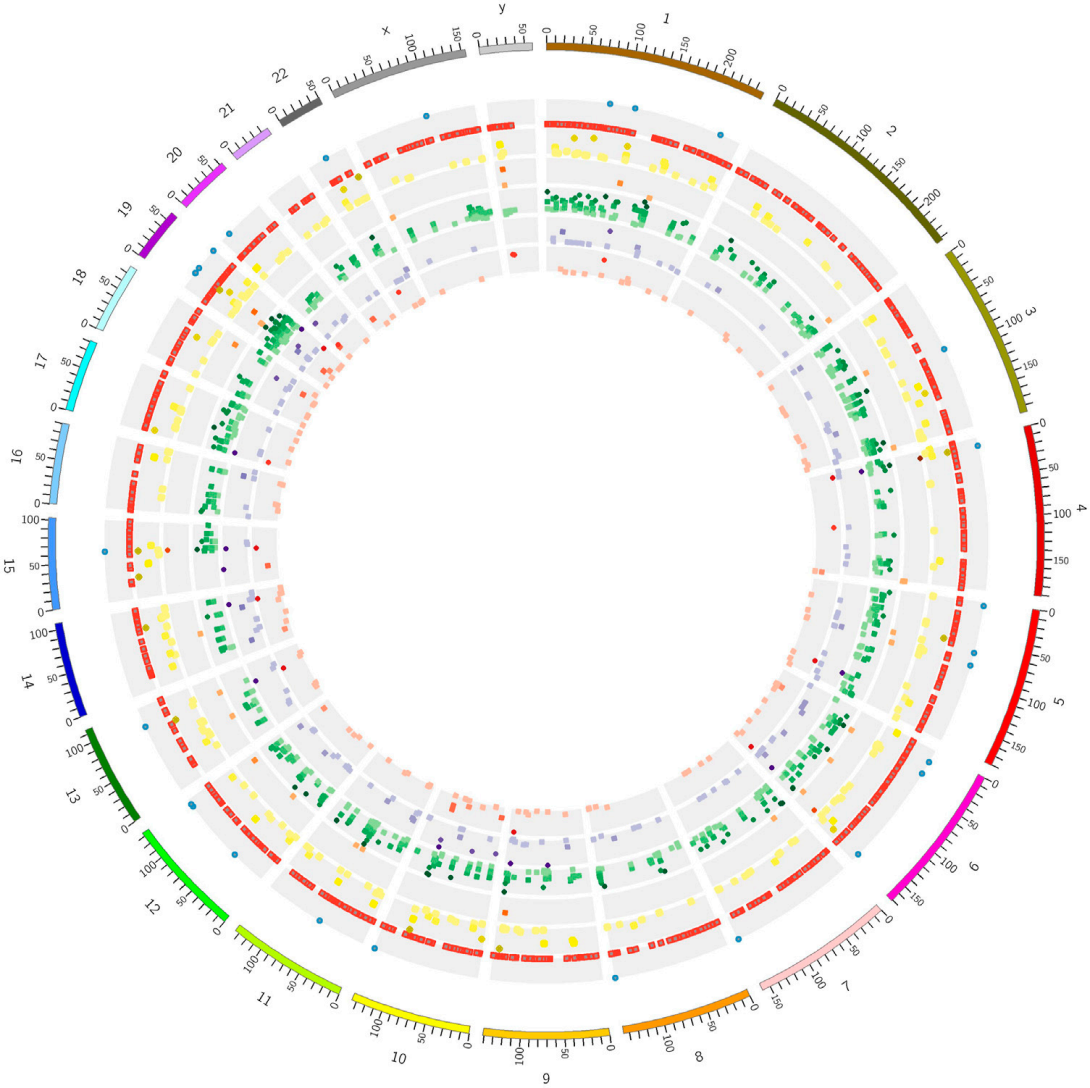
Overview of novel insertions predicted by the tools on the 50 genomes in a circular chromosomal plot. The order of concentric circles from the outside of the plot: circle 1 (blue dots)—known non-reference insertions; circle 2 (red dots)—known reference insertions; circle 3 (yellow): Retroseq predictions; circle 4 (orange): Retroseq plus predictions; circle 5 (green): Steak predictions; circle 5 (purple) ERVcaller predictions, circle 6 (red): Melt predictions. The intensity of the color and the height of each dot in its band is proportional to the number of subjects in whom the insertion is predicted with darker colors and higher position corresponding to a larger number.

Each tool was applied to 50 SR-WGS samples and the results were merged. Table 3 shows the proportion of predicted HERV-K insertions that map to a known HERV locus. Tools with a higher rate of predicted insertions matching to documented previously reported loci are expected to have a higher accuracy as such insertions are more likely to be true positives. It is also important to consider the number of loci given by each tool as they may sacrifice sensitivity to increase the true positive rate. Total number of predictions and overlap with previously reported loci greatly varied across tools (Table 3 and Figure 4) but two of the HERV specific tools (Retroseq+ and ERVcaller) appear to have the highest proportion of predicted insertions that overlapped with previously reported ones. Notably, Steak gave the highest number of predictions and 84% of these results matched to previously documented HERV locations. 39% of Retroseq’s predictions and 52% of Melt’s predictions were previously reported. ERVcaller and Retroseq+ generated sets of predicted loci that greatly matched to previously reported ones (81% and 97.6% respectively) (Kahyo et al., 2017). Mobster was not able to detect any HERV-Ks in this sample. The proportion of HERV-Ks being present in introns, exons and intergenic regions was broadly consistent across tools and in line with results from previous studies (Supplementary Table S1). We also report the frequencies of HERV-K integrations for each tool (Supplementary Table S2).

**TABLE 3:** The “Known Polymorphic” column shows the proportion of predicted HERV-K insertion loci that matched to HERV-Ks from the literature reported to be polymorphic (Supplementary Table S1). “UCSC HERV-K” and “UCSC HERV” columns show the proportion which matched to hg19 reference HERV-Ks and HERVs given by the UCSC table browser (Supplementary File S2, Supplementary File S3). Total percent previously reported shows the proportion of predictions that are present in the polymorphic set or in the UCSC sets. “Total No. predictions” is the total number of predictions given across all 50 genomes. *There is an overlap between the Non-reference polymorphic HERV-Ks and the “UCSC HERV/LTR” set, this explains why the “Total % previously reported” column is less than a sum of these two columns for most tools. Top performing and lowest performing tools are highlighted in blue and red respectively.

	Known polymorphic HERV-Ks (S1)		UCSC HERV-K (S2)	UCSC HERV/LTR (S3)	Total % previously reported*	Total no. predictions
	Reference (%)	Non-reference (%)	Reference (%)	Reference (%)		
Retroseq	0	11	2	31	39%	2,286
Retroseq+	0	64	7	38	97.6%	296
Melt	0.6	26	0	34	52%	638
Steak	0.8	1.7	56	65	84%	13,770
ERVcaller	0	61	6	33	81%	439
Mobster	0	0	0	0	0%	0

**FIGURE 5:**
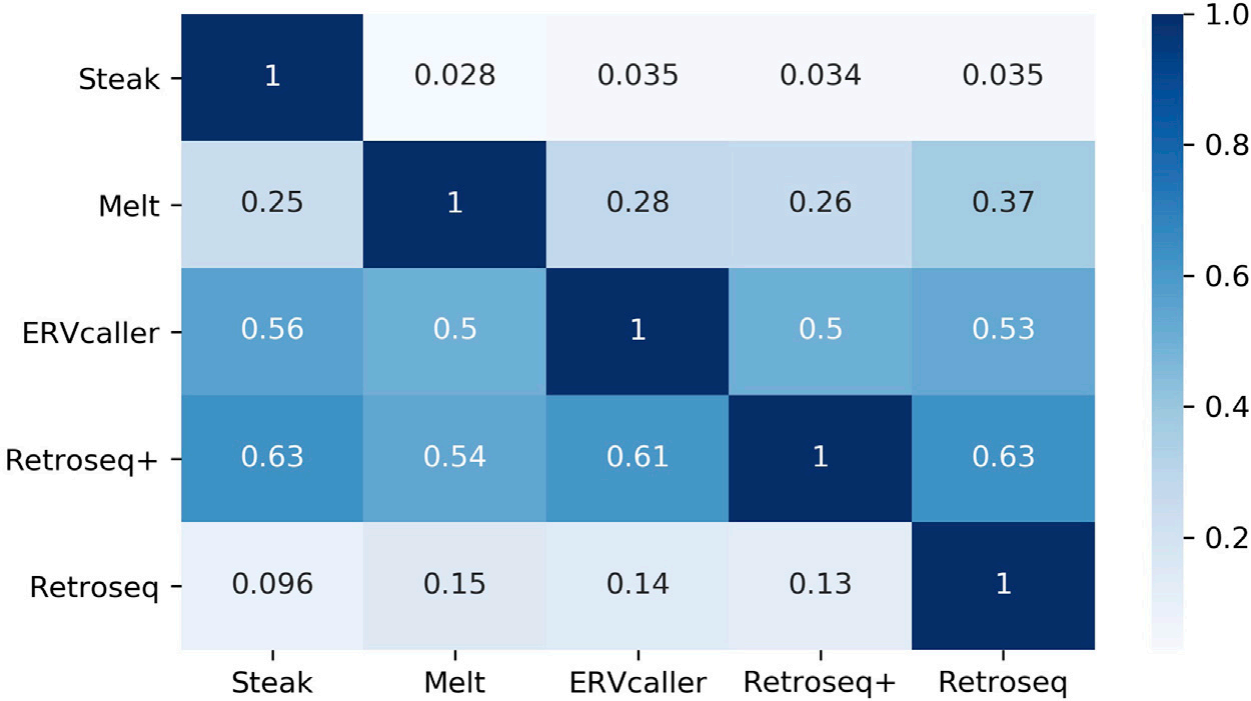
Heatmap reporting the proportion of insertions found by tools on the rows that were also found by the tools on the columns. E.g. the proportion in (Steak, ERVcaller) represents the proportion of Steak calls that were also called by ERVcaller. Therefore, please note that (Steak, ERVcaller) is not equal to (ERVcaller, Steak). Reference HERV-Ks have been filtered out.

The agreement between tools (Figure 5) greatly varied, ranging between 2.8% (proportion of Steak calls that were also called by Melt) and 63% (proportion of Retroseq+ calls that were also called by Steak). The number of insertions predicted ranged between 296 (Retroseq+) and 13,770 (Steak).

### 3.3 Analysis of matching short and long-read sequencing samples

We ran each tool on six SR-WGS samples and used the matching long-read sequencing data for validation (Table 4). Consistently with the other tests, the tools’ performance varied. Generally, the HERV specific tools outperformed the generalist tools in this test, though Retroseq had slightly higher proportions of confirmed calls than ERVcaller. Retroseq+ gave the smallest number of predictions, however, 78% of predicted loci were positive for an LTR5_Hs of length >850 bp in the corresponding long-read sample. We are particularly interested in the larger insertions as they would suggest a complete LTR (968 bases) that will most likely contain regions of biological importance such as the LTR promoters and enhancers. The great majority of the loci predicted by the tools were confirmed to contain ERV sequences in the long-read data. However, considering only the predicted insertions that correctly contained LTR5_Hs (the target HERV-K element), the performance of the tools varied greatly. For example, 78% of insertions called by Retroseq+ were LTR5_Hs, while only 13% of the Melt calls were LTR5_Hs. Most Retroseq+ calls (94%) were >850 bases while a substantial proportion of the loci identified by the other tools were smaller. Moreover, the number of predicted insertions also varied, ranging between 18 (Retroseq+) and 481 (ERVcaller). Notably, Steak identified a large number of long LTR5_Hs insertions but over two-thirds were reference loci and Steak showed a substantially higher precision for reference loci (>77%) than for non-reference loci (41%). Supplementary table 4 shows the proportion of predicted loci for which RepeatMasker reports either HERV-K internal gene sequence or an SVA of at least 50 bps in length. ERVcaller has the highest number of SVA positive loci (61%) while Retroseq+ and Steak have the highest proportion of HERV-K internal gene positive loci (11% and 12% respectively).

**TABLE 4:** This table shows the proportion of results predicted by each tool in the short-read samples, that are positive for a HERV sequence in the associated long-read data. Each column shows the proportion of results that were positive for HERV-K (LTR5_Hs, the targeted HERV subgroup) or a general HERV sequence in the long-read contig data. The results are stratified by the length of the HERV sequence found in the long-read contig data. The Steak results are reported both including and discarding reference HERV loci. Reference HERV loci were removed for all other tools. Top performing and lowest performing tools are highlighted in blue and red respectively.

	LTR5_Hs > 850 bp	LTR5_Hs > 400 bp	LTR5_Hs all	ERV >850 bp	ERV >400 bp	ERV all	Total no. predictions
Retroseq	17%	21%	23%	52%	86%	96%	247
Retroseq+	78%	78%	78%	94%	100%	100%	18
Melt	10%	11%	13%	42%	79%	95%	412
Steak Non-reference	33%	37%	41%	57%	73%	92%	51
Steak ref + non-ref	74%	76%	77%	86%	92%	98%	172
ERVCaller	14%	15%	16%	34%	75%	92%	481
Mobster	NA	NA	NA	NA	NA	NA	NA

### 3.4 CPU usage and time

Finally, the tools were run on a single short-read whole-genome sequencing sample from Project MinE to quantify their memory, CPU and storage usage efficiency. Time, memory and space used were recorded (Table 5). This was achieved using the in-built Slurm HPC scheduling system. All of the tools had a relatively similar CPU time (mean = 3:59 CPU hours) and hard disk usage (mean = 2 GB) with the exception of ERVcaller which had a much higher CPU time (14:17 CPU hours) and used a lot more storage space (87 GB). This contrasts with the results of the original ERVcaller paper which showed that ERVcaller was faster than Retroseq and Melt. A key difference is that, in our test, ERVcaller was run on two CPUs but in the original paper it was run on 12. If the user has a large number of samples, or limited computational resources, ERVcaller may not be appropriate.

**TABLE 5:** This table shows how memory is used by each tool. The CPU time is equal to the number of CPUs * time. MAX VM size is the maximum virtual memory used at any one time by any part of the job. The input file size column reports the size of input sequencing data in the format required by each tool. The Max Temporary Files Size shows the maximum temporary storage required by each tool while running. For tools where there is an option to remove (clean up) temporary files, this option was not used. Lowest performing tool is highlighted in red.

	CPU time	Max vm size (GB)	Input file format/Size (GB)	Max temporary files size (GB)
Retroseq	03:43:01	1.15	BAM/77	2
Retroseq+	04:01:15	1.15	BAM/77	2
Melt	03:23:05	5.10	BAM/77	3
Steak	04:48:35	1.17	SAM/287	<1
ERVcaller	14:17:22	22.39	BAM/77	87

## 4 Discussion

This study compared the performance of six computational tools for detecting HERV loci in whole-genome sequencing data. Three of the tools we tested, ERVcaller, Steak and Retroseq+, were developed to identify exclusively HERVs, while the other three, Retroseq, Mobster and MELT, were designed to identify a broader range of TEs. Our results provided evidence of their highly variable performance across SR-NGS datasets, however, in all experiments HERV specialist tools generally performed better than generalist TE callers in calling HERVs.

The first test involved applying each tool to simulated WGS. In order to simulate potentially realistic (proviral) insertions, we first generated WGS samples using hg19 varying read length and coverage depth. Then we used a copy of hg19 in which we removed a set of known reference HERV loci, for read mapping of the simulated samples and HERV detection. Therefore, this experiment allowed us to assess how read length and coverage depth affect the tools’ performance and to quantify the tools’ precision and sensitivity (Table 3). As expected, the tools performed better on high quality WGS data (32X and 150 bp reads). Steak’s sensitivity was lower than the other tools for detecting insertions in all simulated genomes (≤20%). Although apparently surprising, this result is consistent with another independent evaluation of Steak (Chen and Li, 2019).

Following this, each tool was applied to 50 WGS samples of real individuals from an ALS cohort. This allowed us to quantify the agreement between tools and the proportion of results that matched to known HERV-K loci (Table 2). In this experiment Mobster could not identify any HERV insertions confirming its inability to detect this type of elements as was suggested by the authors in their original benchmarking analysis. The agreement between tools ranged between 3% and 63%, and the number of insertions predicted ranged between 296 (Retroseq+) and 13,770 (Steak). A part of this variability can be explained by the fact that Steak was designed to detect the presence of both reference and non-reference HERV insertions, however, the tools’ accuracy might also contribute substantially. Indeed, although 65% of Steak’s predictions matched reference HERV loci, only 1.7% overlapped with the highly characterized, non-reference, polymorphic loci. Looking at the proportion of insertions that matched the non-reference HERV-Ks previously reported in the literature can inform us about the quality of the predictions made by the tools. We have greater confidence that these known HERV-Ks are true compared to novel HERV-Ks which have not been previously reported or validated.

The tools were also tested on six publicly available genomes that had undergone both long and short-read sequencing (Table 4). Given the length of the long reads (>10 kbs), this dataset allowed us to confirm the insertions called in the short-read data using the long-reads. In this experiment the great majority (>92%) of all insertions predicted by the tools were confirmed HERVs in the long-read data. However, only Retroseq+ insertions were largely (78%) confirmed to be LTR5_Hs (the target HERV-K element), while the other tools showed a lower ability to distinguish between different HERV LTRs (13%–41%).

Finally, the tools were tested on a single WGS sample, and the time, memory and space used for temporary files were recorded. All of the tools had a relatively similar CPU time and hard disk usage with the exception of ERVcaller which had a much higher CPU time (14:17 CPU hours) and used a lot more storage space for temporary files (87 GB). This contrasts with the results of the original ERVcaller paper which showed that ERVcaller was faster than Retroseq and Melt (Chen and Li, 2019).

In conclusion, our analyses showed that tools and protocols developed specifically for the detection of HERV-Ks, such as ERVcaller, Retroseq+, and Steak, generally outperformed generalist tools such as Mobster and MELT. This trend is clearly visible in Supplementary Table S3 that reports an overview of key results across all benchmarking experiments. This finding is consistent with MELT documentation and supported by a recent paper from Niu et al. (Niu et al., 2021). Niu and colleagues found that HERV-K integrations detected by MELT had a 23% false discovery rate (FDR) when tested using PCR, which was a much higher FDR than the other transposable elements. HERV-K insertions in databases based on MELT, including the widely used GNOMAD-SV (Koch, 2020) and the newer HMEID database (Niu et al., 2021), are likely to be unreliable for use in HERV-K focused studies.

Moreover, the experiments highlighted important characteristics of the tools that the users should consider when designing their analysis pipeline: our implementation of the protocol developed by Wildschutte and colleagues (Retroseq+) produced the most reliable predictions but also the smallest number (296 predictions across 50 genomes, Table 2); Steak was the only tool able to comprehensively capture the presence of reference HERVs but its performance was substantially higher on reference HERVs than on non-reference HERVs; ERVcaller and Retroseq showed a good balance between number of detected insertions and their quality, however, their performance greatly varied across experiments. For example, they showed high precision and sensitivity in the simulated data (Table 3), but when applied to real data that is expected to include a large number of other types of insertions (the initial large SR-WGS dataset and the matching short and long-read data, Tables 2, 4), both of them showed high sensitivity but low specificity.

Given that all tools presented strengths and weaknesses, we recommend the users to base their choice on the requirements and objectives of the study and to consider combining multiple tools and a consensus approach if computationally feasible. For example, for rare genetic diseases in which both common polymorphisms and rare disruptive variants contribute to their genetics, such as ALS and other neurodegenerative disorders, one could combine the ability of Steak to call reference HERVs, with one of the other tools that showed a higher performance on non-reference insertions. Moreover, according to the availability of biological samples for wet-lab validation, one might choose a more conservative caller such as Retroseq+ or a more sensitive tool such as ERVcaller.

A limitation of this study is that it is focused on the detection of non-reference HERV insertions and it does not consider HERV annotation. HERV annotation could provide key pieces of information such as HERV family, subtype, location of promoter and enhancer regions, genotype, truncations and other polymorphisms, and whether they have potential for transcription. These are essential for their study and biological interpretation (Grandi et al., 2021; Jia et al., 2022). However, while this type of analysis can be performed for reference HERV loci, it is not possible for non-reference HERV detected in short-read NGS given that this technology does not allow for the characterization of the insertion sequence beyond the read-length.

In interpreting our results, it is important to note that our data may stem from the use of the hg19 reference genome. Results might be slightly different using hg38 as it includes more alternate sequences as well as corrections to sequencing artefacts (Schneider et al., 2017). However, the overarching challenge in calling HERVs remains, regardless of which reference is used, as short-read sequencing presents intrinsic limitations to capture large insertions. This challenge applies to most types of variants larger than some tens of base pairs and consensus approaches have shown potential, e.g. Gnomad SV (Koch, 2020). Long-read sequencing can provide a better solution to the detection of large insertions and its use is on the rise, analyzing short-read sequencing data for large variants is still highly relevant given the great availability of this type of data and its higher per base sequencing resolution.

## Data Availability

The original contributions presented in the study are included in the article and in the Supplementary Material, further inquiries can be directed to the corresponding author. Supplementary materials, including all supplementary tables, figures and the scripts to run the analyses, are available on GitHub: https://github.com/KHP-Informatics/tools_assessment_hervk_SR-WGS.
